# Implantation of a Poly-l-Lactide GCSF-Functionalized Scaffold in a Model of Chronic Myocardial Infarction

**DOI:** 10.1007/s12265-016-9718-9

**Published:** 2017-01-23

**Authors:** Cristiano Spadaccio, Francesco Nappi, Federico De Marco, Pietro Sedati, Chiara Taffon, Antonio Nenna, Anna Crescenzi, Massimo Chello, Marcella Trombetta, Ivancarmine Gambardella, Alberto Rainer

**Affiliations:** 1 Department of Cardiothoracic Surgery, Golden Jubilee National Hospital, Agamemnon Street Clydebank, Glasgow, G81 4DY UK; 2 Institute of Cardiovascular and Medical Sciences, College of Medical, Veterinary and Life Sciences, University of Glasgow, Glasgow, UK; 3 Department of Cardiac Surgery, Centre Cardiologique du Nord de Saint-Denis, Paris, France; 4 Laboratory of Virology, Regina Elena Institute for Cancer Research, Rome, Italy; 5 Department of Imaging and Diagnostics, Università Campus Bio Medico di Roma, Rome, Italy; 6 Department of Pathology, Università Campus Bio Medico di Roma, Rome, Italy; 7 Department of Cardiac Surgery, Università Campus Bio Medico di Roma, Rome, Italy; 8 Tissue Engineering Lab, Università Campus Bio Medico di Roma, Rome, Italy; 9 Department of Cardiac Surgery, Liverpool Heart and Chest Hospital, Liverpool, UK

**Keywords:** Tissue engineering, 3D scaffold, Cardiac graft, Electrospinning, PLLA, GCSF, Myocardial infarction

## Abstract

A previously developed poly-l-lactide scaffold releasing granulocyte colony-stimulating factor (PLLA/GCSF) was tested in a rabbit chronic model of myocardial infarction (MI) as a ventricular patch. Control groups were constituted by healthy, chronic MI and nonfunctionalized PLLA scaffold. PLLA-based electrospun scaffold efficiently integrated into a chronic infarcted myocardium. Functionalization of the biopolymer with GCSF led to increased fibroblast-like vimentin-positive cellular colonization and reduced inflammatory cell infiltration within the micrometric fiber mesh in comparison to nonfunctionalized scaffold; PLLA/GCSF polymer induced an angiogenetic process with a statistically significant increase in the number of neovessels compared to the nonfunctionalized scaffold; PLLA/GCSF implanted at the infarcted zone induced a reorganization of the ECM architecture leading to connective tissue deposition and scar remodeling. These findings were coupled with a reduction in end-systolic and end-diastolic volumes, indicating a preventive effect of the scaffold on ventricular dilation, and an improvement in cardiac performance.

## Introduction

Cardiac tissue damage associated to a myocardial infarction (MI) origins a biological cascade leading to substitution of cardiac muscle with scar formation [1, 2]. In the initial stages characterized by a general inflammatory activation, cardiac cells and stem cells recruited from bone marrow show high proliferation and regenerative potential. However, these compensatory mechanisms lead at the same time to the activation of resident fibroblast resulting in extracellular matrix (ECM) deposition to replace tissue loss and avoid ventricular dilation. Progressive myocardial degeneration with formation of a nonfunctional scar follows, with alteration of the elastic and mechanical properties of the ventricle until pump failure and dysfunction of the conduction system, leading to potential re-entrant circuits and increasing the risk of ventricular tachyarrhythmias, which represent the major cause for death in heart failure patients [2–4]. Recently, granulocyte colony-stimulating factor (GCSF), a growth factor routinely used in clinical hematology and known to mobilize endogenous bone marrow (BM)-derived cells, fueled the interest of scientists because of some direct actions on cardiac cells [5–9].

In the field of TE, the idea of a biomimetic approach based on the simulation of the ECM along with the guidance of resident and stem cell differentiation aided by a growth factor is emerging as a new perspective [10, 11]. The rationale underlying this approach concerned the possibility to exploit the endogenous reparative capabilities of the body and to guide these regenerative resources toward tissue restoration by means of a tailored absorbable material [11, 12]. The importance to concentrate and spatially organize a biological mediator within a 3D ambient, which resembles the native tissue histoarchitecture, has been recently pointed out [10, 13, 14]. Functionalization of the scaffold with growth factors and other biological signals could permit to produce a drug-delivering device providing a structured support to guide cell differentiation and orient tissue regeneration. This idea acquires particular significance in cardiac tissue engineering, in which artificial myocardial patches might be used to prevent geometrical remodeling of the infarcted myocardium and to promote myocardial restoration. In this context, an engineered functionalized myocardium could provide a mechanical mean for ventricular restraint avoiding its dilation. At the same time, it could create a biological ambient suitable to sustain the proliferative capacity of the cells surrounding the infarcted area and a molecular pathway to stimulate cell differentiation [15].

Considering the reported effects of a GCSF cardiac cell proliferation and its beneficial action of the prevention of conductance aberration after MI, we realized a GCSF-releasing polymeric scaffold in poly-l-lactide (PLLA) electrospun fibers and skeletal myoblasts have been cultured in it to obtain a tissue-engineered cardiac graft (TECG) [16]. The electrospinning technique used to manufacture the scaffold allowed for the creation of fibrillary matrix with ultrastructural properties featuring in micrometric scale and thus closely simulating the native ECM histoarchitecture [17]. Skeletal myoblasts seeded in the scaffold acquired morphological characteristics of cardiomyocytes. Cells appeared elongated, and presence of Cx43-positive cellular junctions resembling the gap junctions normally encountered in the myocardium could be observed. These findings, coupled with the expression of cardiac specific isoform of troponin-I, reliably indicate the in vitro promotion of a cardiomyocyte-like profile in this GCSF-functionalized scaffold [16].

Considering the demonstration of the in vitro effectiveness of the functionalized biopolymer previously developed, in the present study, we sought to evaluate the effect of the implantation of a GCSF-releasing microstructured biomaterial, prepared as a ventricular patch, in an animal model of chronic myocardial infarction.

## Materials and Methods

### Scaffold Preparation and Characterization

GCSF-releasing PLLA scaffolds (PLLA/GCSF sample) were prepared by electrospinning as previously described [18]. Briefly, GCSF (Filgrastim, 30 MUI/mL, sodium salt, Amgen, Thousand Oaks, CA) was combined to a 13% *w*/*w* PLLA (Sigma-Aldrich, St. Louis, MO) polymer solution at a concentration of 250 UI/g, corresponding to the dosage routinely used in literature and in clinical settings [19]. Procedural parameters [16] and sterilization techniques [20] are described elsewhere.

Pristine PLLA scaffolds (PLLA/CTRL sample) were manufactured using the same experimental conditions to obtain a control for biological experiments.

Mechanical and microstructural characterization of the materials, as well as determination of the drug release profile, have been reported elsewhere [18] and performed by means of field emission scanning electron microscopy (FE-SEM), longitudinal uniaxial testing according to Sell et al. with peak stress (PS) and strain at failure (SF) evaluation [21], and specific ELISA assay for GCSF quantitation.

### Experimental Protocol

1. General Overview

Estimation of the total number of rabbits (replicates per group) was performed via an inverse power analysis by calculating the number required to detect a significant difference in biological endpoints between the control and the experimental groups. For this project, data generated by Tan et al. evaluating the effectiveness of a MSC-seeded intestinal submucosa in a model of rabbit myocardial infarction in terms of left ventricular function and tissutal capillary density in comparison to control were used [22]. This calculation gave a sample size of four animal surgeries per condition tested. Considering 30% mortality and four experimental groups, a total of 24 adult male New Zealand white rabbits weighting 1.8–2.1 kg were purchased (Charles River Laboratory) and housed under controlled conditions and normal diet for 3 weeks before experimentation.

Therefore, total animals were randomly assigned to four groups. Group 1 was made of four healthy control (HEALTHY); group 2 consisted of six animals to be used as a chronic MI control group (MI/CTRL); group 3 was made of seven animals that would receive a pristine PLLA (PLLA/CTRL) patch; and group 4 was made of seven animals that would undergo PLLA-GCSF-functionalized patch (PLLA/GCSF) implantation. As discussed above, patches were planned to be implanted 4 weeks after MI induction. Numerosity of each group was defined at the beginning of the study protocol to ensure that, even in case of adverse events, an adequate number of subjects completed the protocol in each group.

Study design consisted in a first phase of creation of a chronicized localized MI through coronary permanent ligation via left thoracotomy followed by a second phase, 4 weeks after MI, of implantation of a GCSF-releasing poly-l-lactic acid microstructured patch through median sternotomy. Only animals developing a left ventricular dysfunction with ejection fraction inferior to 35% gained access to the second phase. Timing for second surgery was defined according to the reported findings of stabilization of cardiac remodeling process [23] and achievement of a histological plateau in the infarcted myocardium 4 weeks after MI induction [24]. To control for potential biases arising from the use of the biomaterial, results were compared also to a nonfunctionalized, pristine PLLA patch.

Blood test and imaging and functional assessment through echocardiography and CT scans were performed at baseline, 4 weeks after MI induction and 2 weeks following patch implantation before study termination. All procedures, care, and handling of the animals were reviewed and approved by the Institutional Animal Care and Use Committee of the Regina Elena Institute.

### Operative Procedures

1. Anesthesia

An optimized anesthesia protocol was selected in order to guarantee stabilization of cardiovascular function during the open chest procedure [25]. Anesthesia was inducted by intramuscular (i.m.) administration of ketamine hydrochloride (25 mg/kg of body weight) and xylazine hydrochloride (15 mg/kg). After disappearance of the pedal reflex in the hindlimbs, rabbits were placed on a warming operative platform (37 °C) in the supine position. A 23-gauge vascular access was obtained through the marginal vein of the ear. The hair on the anterior and lateral chest wall and on the anterior cervical region was trimmed with an electric clipper. The skin leads of a custom-built electrocardiographic apparatus were attached on both fore limbs and on the left hind limb, and the electrocardiogram was monitored. Before skin incision, one dose of enorfloxacin (100 mg/kg) was administered intramuscularly for prophylaxis against surgical infection. Intravenous anesthesia with propofol (0.6 mg kg^−1^ min^−1^) and fentanyl (0.48 μg kg^−1^ min^−1^) was given and used as maintenance. The trachea was cannulated for artificial respiration. Positive pressure artificial respiration was started immediately with room air with the use of a stroke volume of 20 mL/kg and at a rate of 55 strokes/min as previously described [26] (Harvard Apparatus, Holliston, MA).

2. Myocardial Infarction Protocol

Surgery was performed using aseptic techniques with sterile instruments. The skin of the anterior and lateral chest wall was aseptically prepped with a povidone-iodine solution. To induce reliable MI, studies of Podesser et al. demonstrating the interindividual variability of rabbit coronary anatomy and the differences in regional myocardial perfusion distribution [27] together with protocols developed by Lee et al. to obtain a reproducible myocardial damage according to the ramification pattern of the coronary arteries [28] were used. Briefly, a left thoracotomy at the fourth intercostal space was performed. An incision was made at the cardiac sac to expose the myocardial ventricular wall, and the heart was gently exteriorized by pericardial suspension 2-0 silk sutures; the left coronary artery was then identified by lifting the ventricular wall upward and the coronary ramification pattern evaluated. According to the bifurcation or trifurcation asset found, the major branch supplying blood to the left ventricle and cardiac apex was then ligated at a defined site between the starting point of the major branch and the cardiac apex using a 6-0 monofilament polypropylene suture (Prolene™, Ethicon, Johnson & Johnson, Somerville, NJ, USA) as described by Lee et al. [28] The ligated artery should have corresponded to the posterolateral or lateral branch of the left coronary artery. Myocardial ischemia was confirmed by ST-segment elevation on the ECG and regional cyanosis and/or bulging of the myocardial surface. Lidocaine 1 mg/kg was given intravenously 2 min before and immediately after ligation to prevent ventricular arrhythmias. Left pleural drainage was posed in place applying negative pressure. Interrupted 2-0 resorbable suture was used to approximate the ribs. The muscle layer was reformed using semi-interrupted 3-0 sutures to form a complete seal. The skin was closed with semi-interrupted 4-0 Nylon sutures. A dab of povidone-iodine solution was placed on the suture line. After closure of the chest, the animals were allowed to recover for 30 min on a warming pad and were weaned from artificial ventilation. The surgical procedure was completed within 30 min after the initiation of anesthesia. When responsive to stimuli and able to maintain an upright posture, rabbits were returned to the home cage and analgesia initiated with buprenorphine (0.5 mg/kg) and paracetamol (1 mg/kg).

3. Patch Implantation

Four weeks after MI induction, rabbits underwent similar anesthetic protocol and were placed in supine position and the chest aseptically prepped with a povidone-iodine solution. One percent lidocaine was injected into the subcutaneous area overlying the sternum to enhance analgesia and to prevent the occurrence of myocardial arrhythmia. After a midline muscle and skin incision was made over the sternum, the xiphoid process was carefully detached from the sternal part of the diaphragm. A median sternotomy was then performed; the median incision went straight down the xiphoid process toward the jugular notch of the sternum exactly along the midline of the sternum so that injury to the parietal pleura was avoided. Sternal retractors were then used to spread the sternal edges and maintain surgical exposure. Adhering tissue around the myocardium was carefully removed, and the infarcted region was identified at the level of previous Prolene suture. Infarcted zone was scarified, and PLLA or PLLA/GCSF patch was sutured within the injured myocardium with 7-0 Prolene suture. Group MI/CTRL underwent median sternotomy and all the procedures without patch implantation. One percent lidocaine was given intravenously before and after patch implantation. Chest drainages were placed if necessary, and sternum was closed with four interrupted sutures using 1-0 Vicryl (Ethicon) suture with a taper point needle in order not to damage to the internal thoracic arteries and veins. Muscle layers and skin were then closed with continuous suture using 4-0 nylon with a cutting needle. The rabbits were allowed to recover and were weaned from artificial ventilation. After complete recovery from anesthesia, analgesia protocol with buprenorphine(0.5 mg/kg) and paracetamol (1 mg/kg) was initiated.

### Functional Assessment

1. Echocardiography

Echocardiographic evaluation was performed at baseline, 4 weeks after the induction of MI, and at the end of the study protocol in each subject. Functional assessment was achieved by echocardiography, performed by a blinded investigator on control and treatment groups using a Sequoia C256 system (Acuson, Mountain View, CA, USA) equipped with a 6-MHz linear-array transducer (15L8). Standard 2D and M-mode transthoracic images were recorded at the level of the papillary muscles. All measurements were performed according to the American Society for Echocardiology leading-edge technique and averaged on three consecutive cardiac cycles. Regional wall motion, post-ischemic papillary muscle dysfunction with mitral valve insufficiency, and ventricular diameters and volumes were determined. End-diastolic diameter (EDD) and end-systolic diameter (ESD) and associated volumes were measured; fraction of shortening (FS) and ejection fraction (EF) was calculated by ad hoc software (Acuson, Sequoia, Mountain View, CA, USA).

2. Computerized Tomographic Angiography

Computerized tomographic angiography (CTA, Siemens AG, Erlangen, Germany) was performed on all groups at baseline and in all eligible subjects 4 weeks after MI induction and 2 weeks following patch implantation or control procedure. Rabbits were anesthetized with midazolam at a dose of 2 mg/kg intramuscular. ECG gating was achieved, iodinated contrast medium was injected in the marginal ear vein, and cardiac CT scans were obtained and reformatted in 3D using maximum intensity projection (MIP) and volume rendering algorithms. Ventricular volumes have been calculated by means of Circulation Leonardo software (Siemens) according to perpendicular axes of reconstruction. Left ventricular end-diastolic volume (EDV), left ventricular end-systolic volume (ESV), EF, cardiac output (CO), stroke volume (SV), and left ventricle mass (LVM) were measured by two independent blinded observers, and the mean for each pair of observations was recorded. Healthy subjects were used as controls for pairwise comparisons.

3. Histopathological Examination

Two weeks following patch implantation (for PLLA/CTRL and PLLA/GCSF groups) or control surgical procedure (for MI/CTRL group), rabbits were humanely sacrificed and the heart was exposed by median sternotomy. Heparin (2000 IU) was administered intravenously, and the hearts were quickly excised with the aortic root and retrogradely perfused with heparinized saline. The hearts were cut into equal transverse blocks from apex to base. Samples obtained were fixed in 4% paraformaldehyde and embedded in paraffin. Progressive serial sections (6 μm thick) were cut from paraffin-embedded blocks and used for hematoxylin/eosin (H/E) staining, Masson’s trichrome staining, silver staining (Bio-Optica, Milan), and immunohistochemistry for vimentin, CD68, and CD31. The number of microvessels (diameter < 20 μm) in the infarcted area were counted in paraffin-embedded sections with CD31 staining under light microscopy (magnification ×400). Five high-power fields in the infarcted area were randomly chosen, and microvessels were counted in each field. Vascular density was expressed as the average number of microvessels per unit area (0.2 mm^2^) [29]. Similarly, differential cell count for inflammatory (CD68+) cells and noninflammatory fibroblast-like vimentin-positive cells have been performed according to Peng et al. [30] and Odorfer et al. [31]. Myocardial infarction size was performed 4 weeks after MI induction and at the end of the study as previously described [32, 33].

### Statistical Analysis

Data were processed using SPSS release 20.0 for Windows (SPSS, Chicago, IL). Data are reported as means ± standard deviation (SD). One-way ANOVA was used to compare groups with different treatments, followed by multiple pairwise comparison procedure (Tukey test) with *P* values less than 0.01 considered as significance threshold. Repeated measures ANOVA and two-way ANOVA were performed to compare between the 4- and 6-week results between groups. *P* values less than 0.05 were considered significant.

## Results

### Scaffold Characterization

GCSF-releasing PLLA patch have been fully characterized as for their chemical, biological, and mechanical properties in our previous work. Briefly, FE-SEM characterization of the electrospun materials revealed a homogeneous distribution of porous fibers with an average diameter of 1.30 ± 0.40 μm for PLLA/CTRL and PLLA/GCSF samples. This fiber morphology could represent a suitable environment for cell culturing, as simulating the structure of native extracellular matrix, and potentially provide similar support to cell growth and differentiation. The stress-strain profile showed a bell-shaped curve with a peak stress quantified at 0.188 ± 0.011 MPa and a strain at failure of 0.319 ± 0.016. GCSF release curve showed an initial burst, followed by a sustained release at a much slower rate. Up to 35% of the loaded GCSF was released at 1 week. Nontoxicity of the scaffold was confirmed by preserved cell viability with an increase in cell proliferation rate in a colony of C2C12 myoblasts seeded into a GCSF-releasing patch. Efficiency was testified by the induction of significant cell changes in morphology, biological phenotype, and expected expression of cell markers (CX43) within the GCSF-releasing patch in the experiments previously cited [16].

### Mortalities

Six of the 24 rabbits died within 4 weeks after left anterior descending artery ligation and MI induction (1 in group 2, 2 in group 3, and 3 in group 4), and 2 rabbits were excluded after the first echocardiography examination because their EF was above 35% (1 in group 2 and 1 in group 3). The remaining animals of groups 3 and 4 underwent patch implantation according to study protocol. Therefore, each experimental group was made up of four animals.

### Echocardiography

After MI induction, all the subjects developed mitral insufficiency due to posterior-lateral papillary muscle dysfunction consequent to MI. Additionally, general LV hypokinesia was noted along with left atrial enlargement. Hypokinesia of the superior portion of the interventricular septum was noted, and signs of right ventricular overload were detected in some animals, but no evidence of significant tricuspid regurgitation was demonstrated. Apart from two subjects with EF above 35%, which were subsequently excluded from the analysis, EF in the other subjects was below 35% (mean 30 ± 3%).

**Fig. 1:**
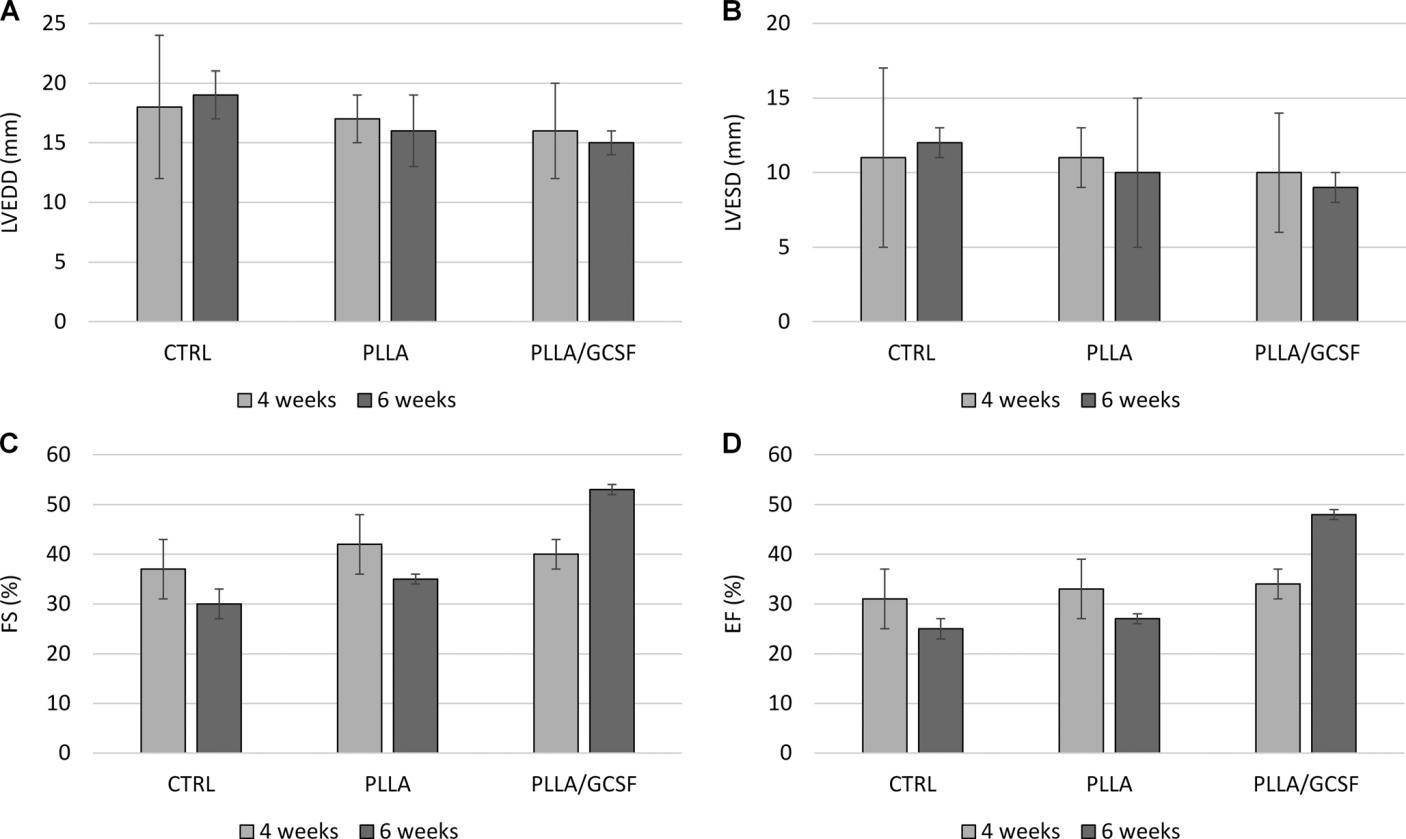
Echocardiographic evaluation of LVEDD (**a**), LVESD (**b**), FS (**c**), and EF (**d**) after 4 weeks (*light gray*) and 6 weeks (*dark gray*) after induced myocardial infarction; the latter corresponds to 2 weeks after patch implantation. *P* values are shown in Table 1

Table 1 and Fig. 1 show echocardiographic findings 4 and 6 weeks post-MI, the latter corresponds to 2 weeks after patch implantation; both EDD and ESD significantly improved in the PLLA/GCSF group compared to MI/CTRL and PLLA/CTRL groups. ESD increased over time in the control and PLLA group indicating an underlying process of LV dilation and post-MI remodeling. In the PLLA/GCSF group, a significant decrease in the ESD was noted 2 weeks after implantation. These data were coupled with a statistically significant improvement at 6 weeks’ control of both FS and EF in the PLLA/GCSF group compared to both MI/CTRL and PLLA/CTRL group.

**Table 1:** Echocardiographic findings 4 and 6 weeks post-myocardial infarction (corresponds to 2 weeks after patch implantation), with *P* values referring to the difference with PLLA/GCSF group

Parameter	Group	4 weeks	6 weeks	*P* (within group)
LVEDD (mm)	MI/CTRL	18 ± 6, *P* = 0.599	19 ± 2, *P* = 0.011	0.762
	PLLA/CTRL	17 ± 2, *P* = 0.670	16 ± 3, *P* = 0.550	0.599
	PLLA/GCSF	16 ± 4	15 ± 1	0.645
LVESD (mm)	MI/CTRL	11 ± 4, *P* = 0.703	12 ± 1, *P* = 0.005	0.645
	PLLA/CTRL	11 ± 1, *P* = 0.550	10 ± 5, *P* = 0.708	0.708
	PLLA/GCSF	10 ± 3	9 ± 1	0.550
LVEDV (mL)	MI/CTRL	10.9 ± 1.2, *P* = 0.975	11.4 ± 1.3, *P* = 0.070	0.592
	PLLA/CTRL	10.8 ± 1.3, *P* = 0.914	11.5 ± 1.6, *P* = 0.090	0.522
	PLLA/GCSF	10.9 ± 1.2	9.6 ± 1.0	0.147
LVESV (mL)	MI/CTRL	7.5 ± 1.3, *P* = 0.755	8.5 ± 1.2, *P* = 0.005	0.301
	PLLA/CTRL	7.2 ± 1.2, *P* = 0.958	8.4 ± 1.3, *P* = 0.007	0.223
	PLLA/GCSF	7.2 ± 1.3	5.0 ± 1.1	0.042
FS (%)	MI/CTRL	37 ± 6, *P* = 0.405	30 ± 3, *P* < 0.001	0.082
	PLLA/CTRL	42 ± 6, *P* = 0.573	35 ± 1, *P* < 0.001	0.061
	PLLA/GCSF	40 ± 3	53 ± 1	<0.001
EF(%)	MI/CTRL	31 ± 2, *P* = 0.078	25 ± 2, *P* < 0.001	0.005
	PLLA/CTRL	33 ± 2, *P* = 0.506	27 ± 1, *P* < 0.001	0.001
	PLLA/GCSF	34 ± 2	48 ± 1	<0.001

*P* values in the separate column represents the statistical significance of the within group difference

*LVEDD* left ventricle end diastolic diameter, *LVESD* left ventricle end systolic diameter, *FS* fraction of shortening, *EF* ejection fraction

### Computerized Tomographic Angiography

**Fig. 2:**
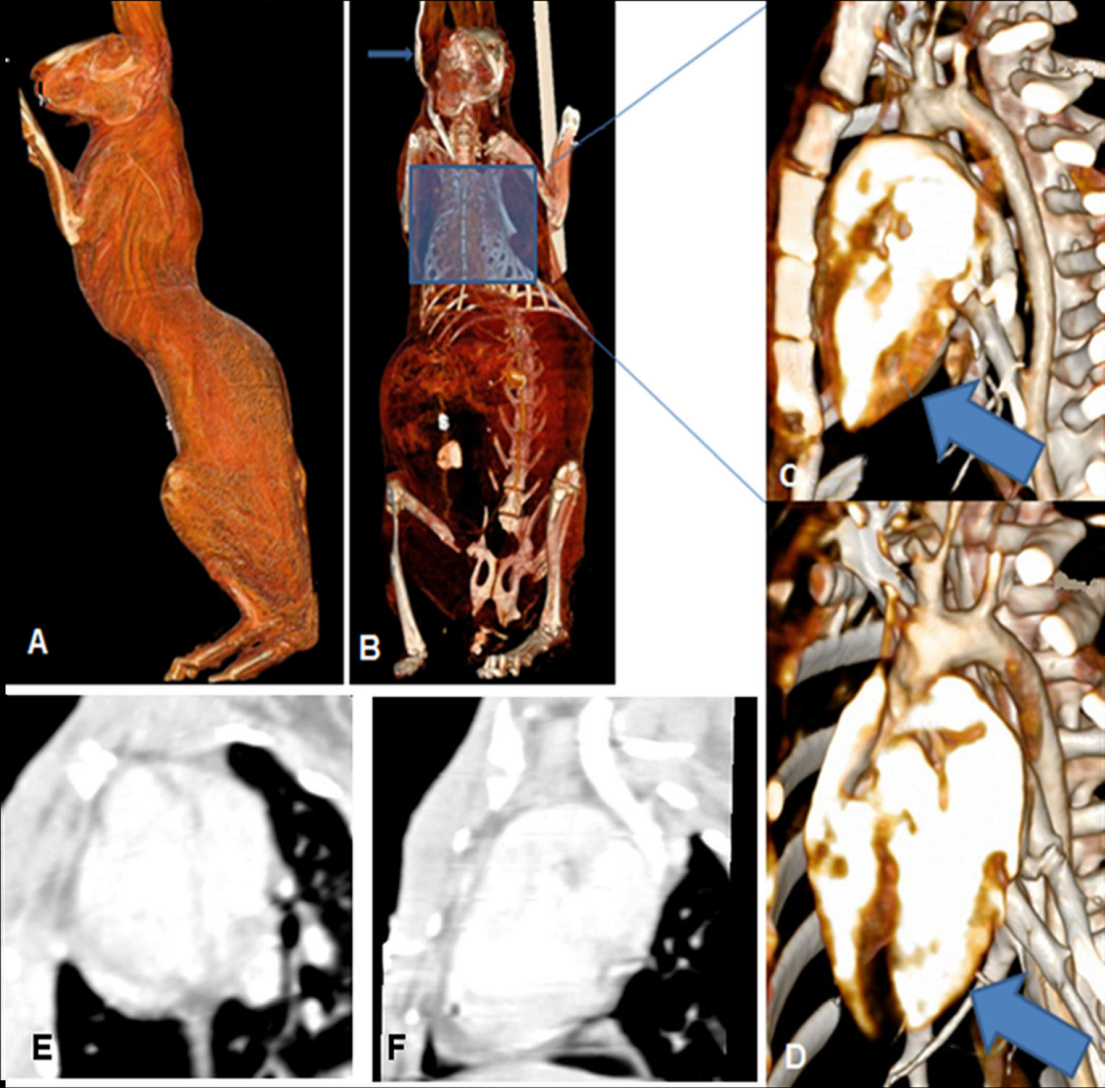
Volume-rendering CT reconstructions. Positioning of the subject (**a**, **b**). Normal myocardial wall thickness (*arrow*) before coronary ligature (**c**). After posterolateral artery ligature, ischemic area shows a marked reduction in ventricular thickness (**d**).Short axis (**e**) and long axis (**f**) scans showing impaired perfusion in the apex and in the lateral wall

A reduction in myocardial wall thickness could be observed according to the localization of the induced MI (Fig. 2).

**Fig. 3:**
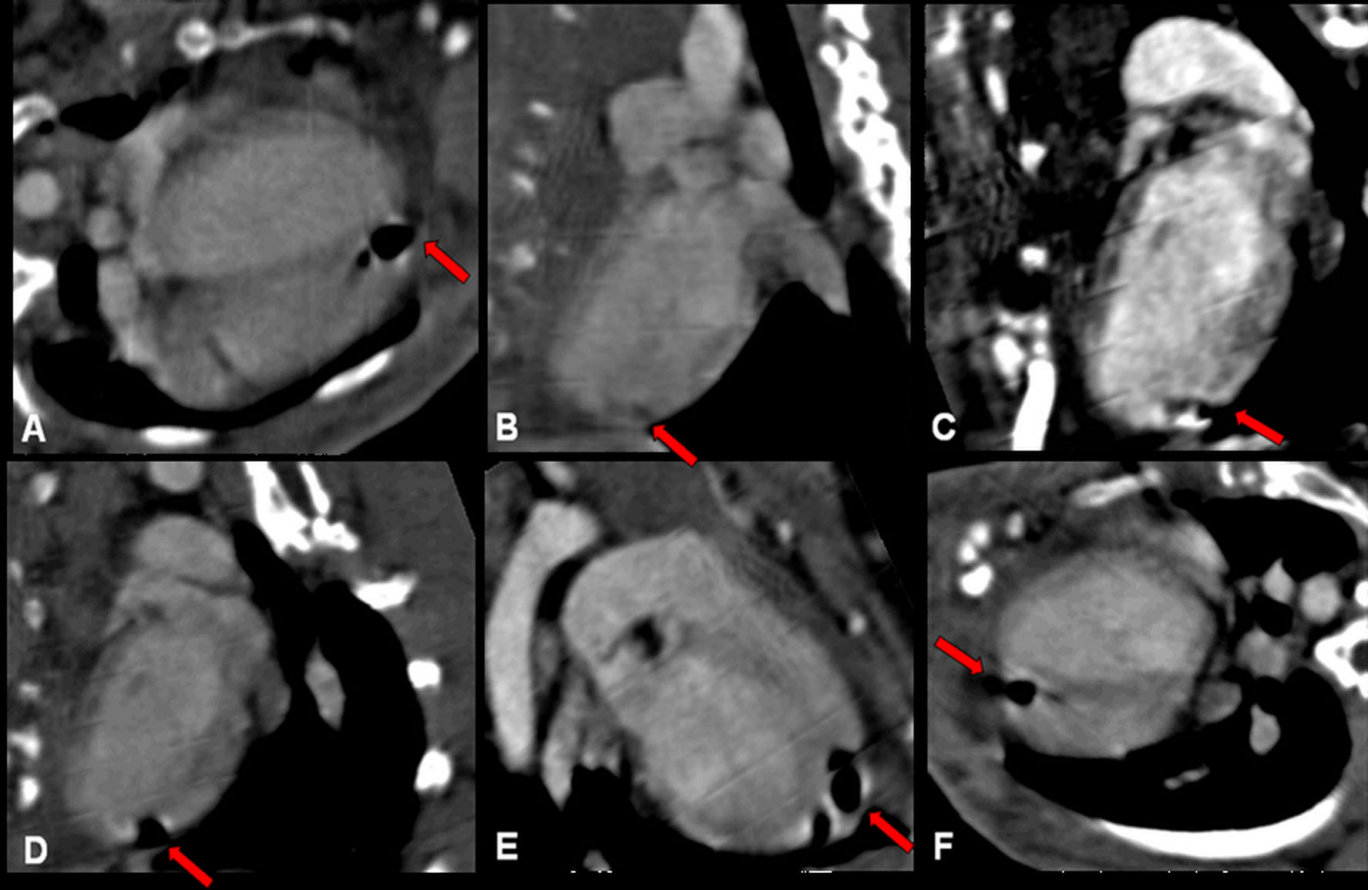
PLLA/CTRL group: long axis (**a**–**e**) and short axis (**f**) planar reconstructions from CT scans. Ischemic area is characterized by a small area of tissue loss. Patch is radiolucent and is indicated by *red arrows*

**Fig. 4:**
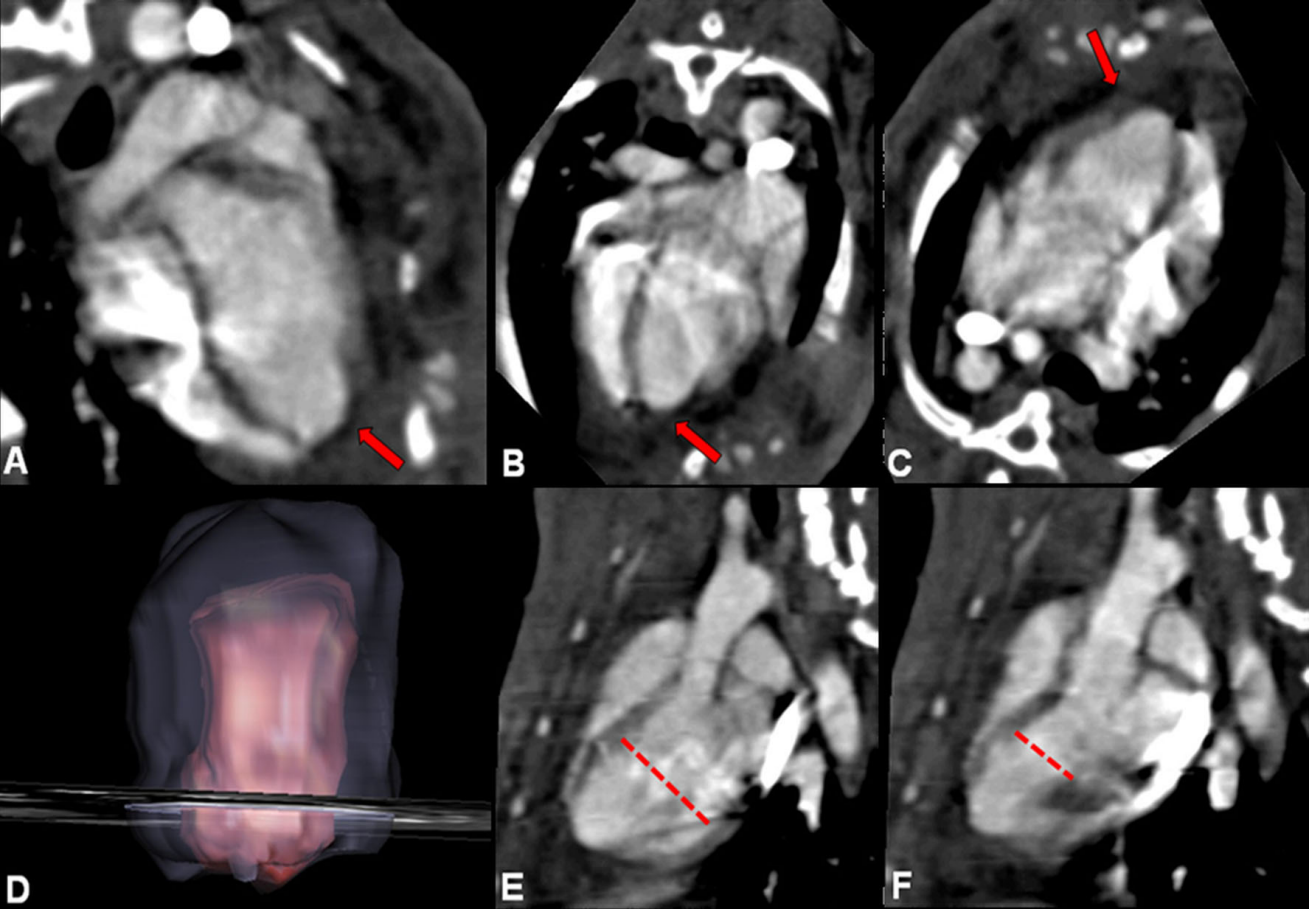
PLLA/GCSF group: planar (**a**–**c**), volume rendering (**d**), end-diastolic long axis (**e**), and end-systolic long axis (**f**) reconstructions from CT scans. Ischemic area is characterized by a small area of tissue loss. Patch is radiolucent and is indicated *by red arrows*. Ventricular diameter measurements were performed using these sequences (*dashed lines*)

In the groups implanted with PLLA or PLLA/GCSF patches, the biomaterial could be detected as a radiotransparent region lying over the area of wall thinning (Figs. 3 and 4).

**Fig. 5:**
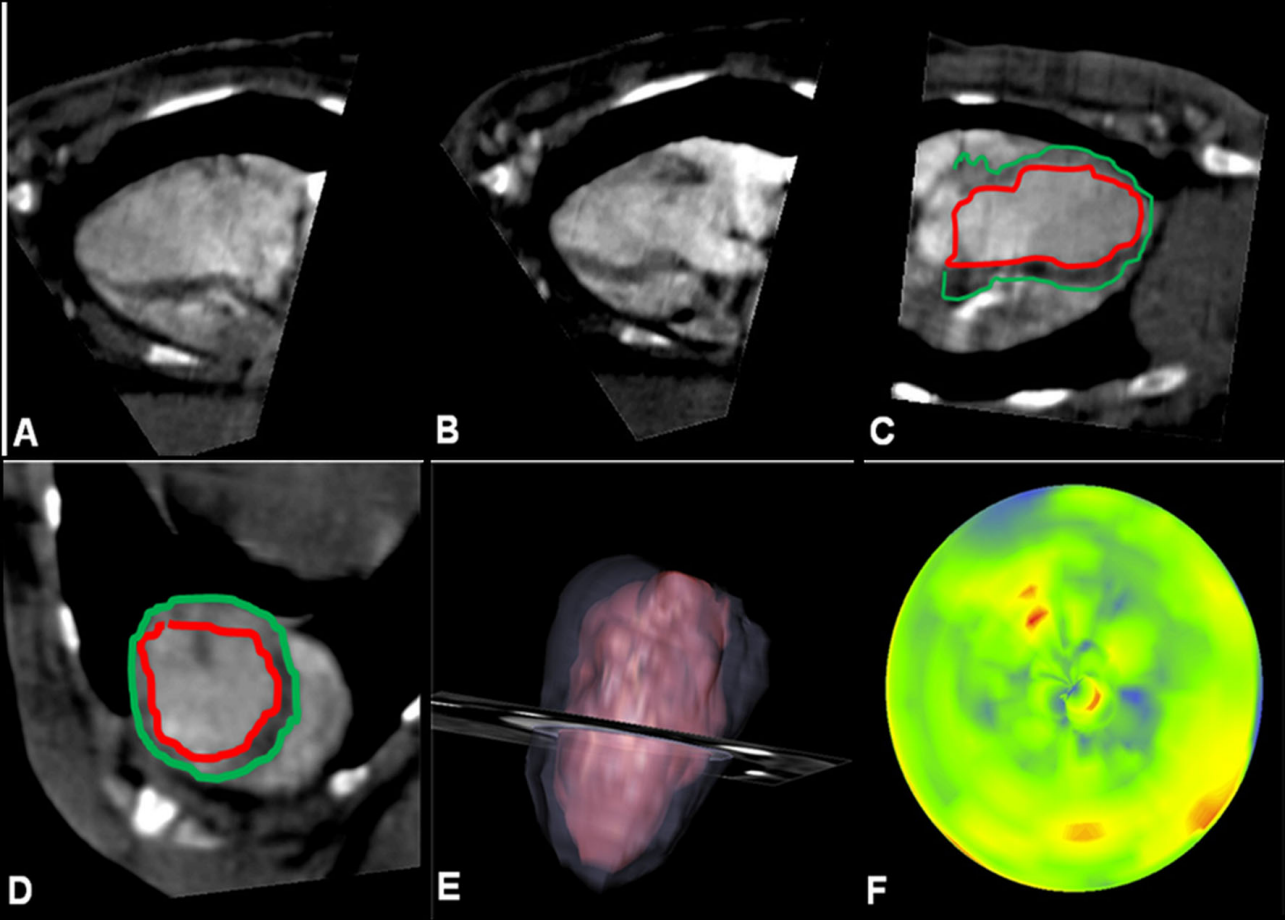
Exemplificative ECG-gated CT morphovolumetric analysis of a MI/CTRL subject. Diastolic (**a**) and systolic (**b**) acquisition. Definition of inner and outer margins in long axis (**c**) and in short axis (**d**) view, followed by reconstruction (**e**). Graphical representation of regional wall motion with colorimetric scale, where *dark areas* indicate reduced kinesis (**f**)

Measurement of EDD and ESD confirmed the findings obtained at echocardiography with a significant reduction in both the diameters 6 weeks after PLLA/GCSF patch implantation. By synchronization with cardiac cycle, ECG-gated images were obtained showing a topographical map of myocardial wall thickening and regional kinesis (Fig. 5).

EDV, ESV, EF, CO, SV, and LVM could be calculated and are shown in Table 2. PLLA/GCSF group showed a significant improvement of EDV with an EF of 43 ± 0.1%, and the calculated CO improved to 0.51 ± 0.5 L/min. LVM was significantly improved in the PLLA/GCSF group compared to MI/CTRL group and PLLA/CTRL group.

**Table 2:** Left ventricle end diastolic volume (LVEDV), left ventricle end systolic volume (LVESV), ejection fraction (EF), cardiac output (CO), stroke volume (SV), and left ventricle mass (LVM) measured with CT scan, 6 weeks post-myocardial infarction

	HEALTHY	MI/CTRL	PLLA/CTRL	PLLA/GCSF
EDV (mL)	8 ± 0.3	10 ± 0.6	11 ± 0.5	9 ± 0.3
	(*P* = 0.003)	(*P* = 0.025)	(*P* < 0.001)	
ESV (mL)	3 ± 0.2	8 ± 0.3	8 ± 0.4	5 ± 0.5
	(*P* < 0.001)	(*P* < 0.001)	(*P* < 0.001)	
EF (%)	63 ± 0.3	33 ± 0.4	35 ± 0.2	43 ± 0.1
	(*P* < 0.001)	(*P* < 0.001)	(*P* < 0.001)	
SV (mL)	5 ± 0.6	2 ± 0.2	3 ± 0.3	4 ± 0.4
	(*P* = 0.032)	(*P* < 0.001)	(*P* = 0.007)	
CO (L/min)	0.66 ± 0.5	0.25 ± 0.6	0.37 ± 0.8	0.51 ± 0.5
	(*P* = 0.686)	(*P* = 0.546)	(*P* = 0.777)	
LVM (g)	9 ± 0.3	5 ± 0.2	6 ± 0.4	7 ± 0.6
	(*P* = 0.001)	(*P* = 0.007)	(*P* = 0.032)	

*P* values refer to the difference with PLLA/GCSF group

### Histology

**Fig. 6:**
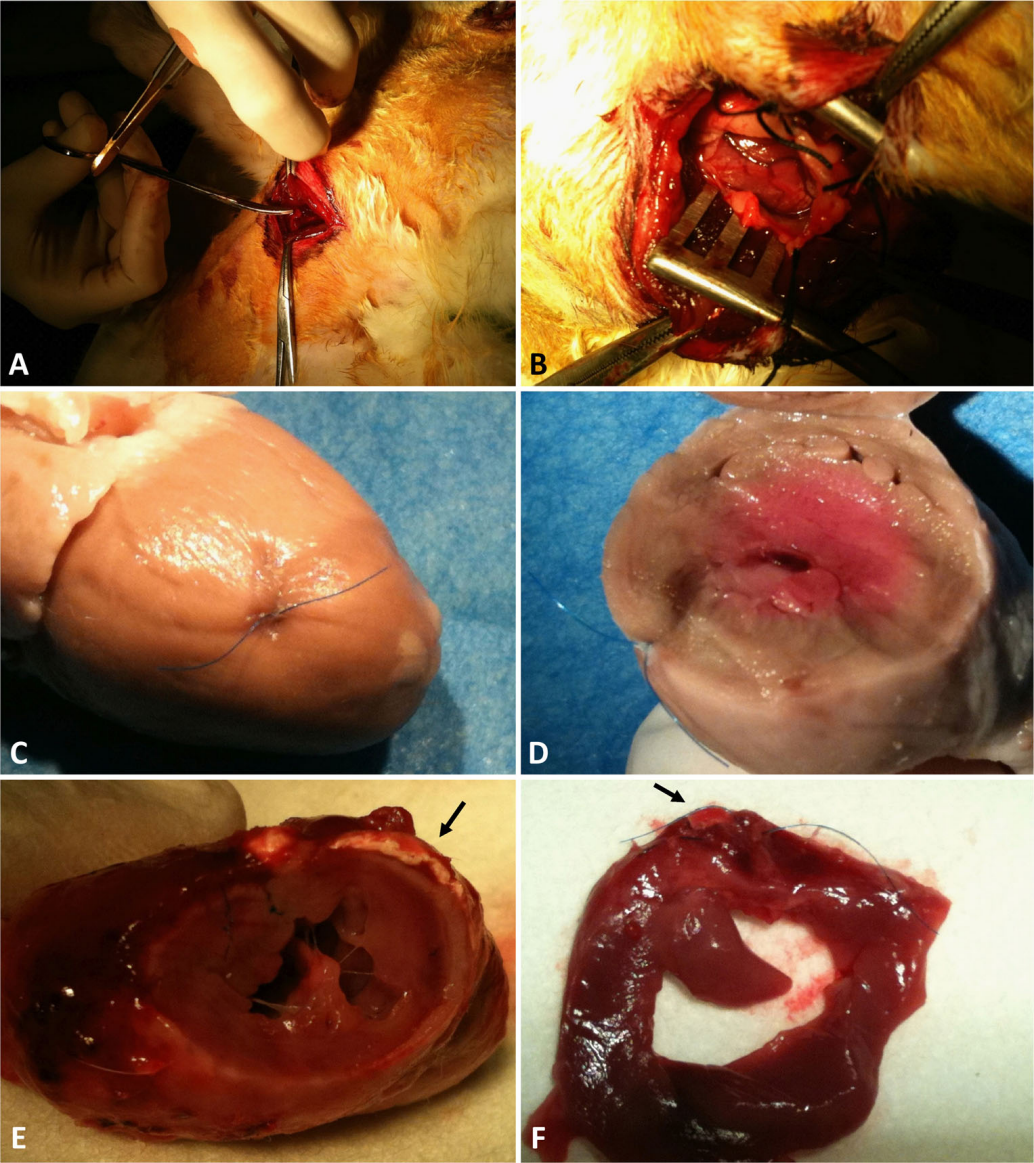
Heart exposure through left thoracotomy (**a**), with vessel identification after pericardial suspension (**b**). Macroscopic evaluation of the infarcted heart in MI/CTRL group (**c**, **d**). PLLA/GCSF patch (*arrow*) is integrated in myocardial tissue, visible as a white sheet (**e**, **f**)

**Fig. 7:**
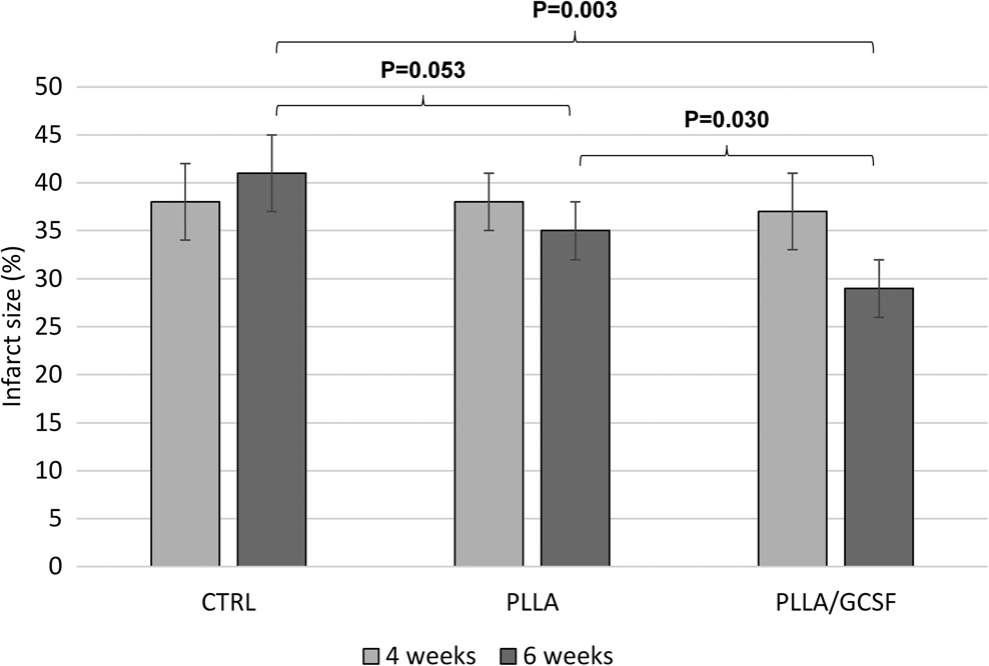
Myocardial infarction size among groups after 4 weeks (*light gray*) and 6 weeks (*dark gray*) after induced myocardial infarction. Myocardial infarction size is expressed as the percentage of the left ventricular myocardial volume

Intraoperative findings and macroscopic evaluation are shown in Fig. 6. Infarcted area is clearly visible, and patch appears to be juxtaposed to the myocardium. Myocardial infarction size measurement 4 weeks after MI induction and at the end of the study are reported in Fig. 7. No statitistically significant difference among groups could be observed soon after MI induction (at 4 months) indicating the achievement of a similar baseline extension of the infaction across the groups in terms of infarction size and transmurality. However, at the end of the study, the physiological process of myocardial expansion occurring in the MI/CTRL group seemed to have been attenuatd or counteracted in both the groups treated with patch implantation, although the PLLA/GCSF group showed the most significant reduction in MI scar sized. This finding was coupled with the previosly mentioned improvement in EDV and LVEED indicating a lesser extent of post-MI ventricular dilation.

**Fig. 8:**
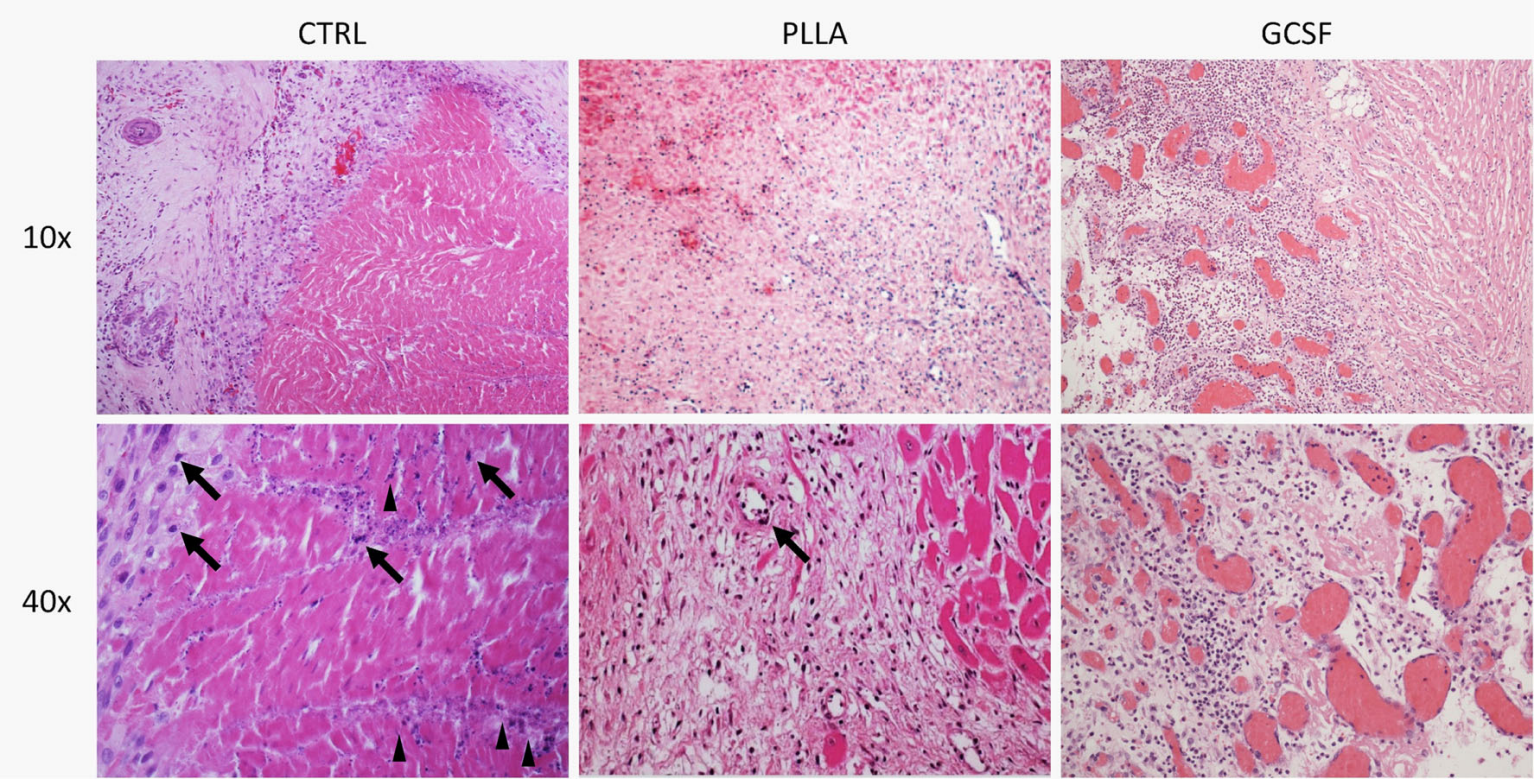
H/E staining of the infarcted area, with ×10 (*upper panel*) and ×40 magnification (*lower panel*). Myocardial tissue showed typical features of chronic infarction with nuclear picnosis (*black arrows*) and intercellular patchy calcifications (*arrowheads*)

**Fig. 9:**
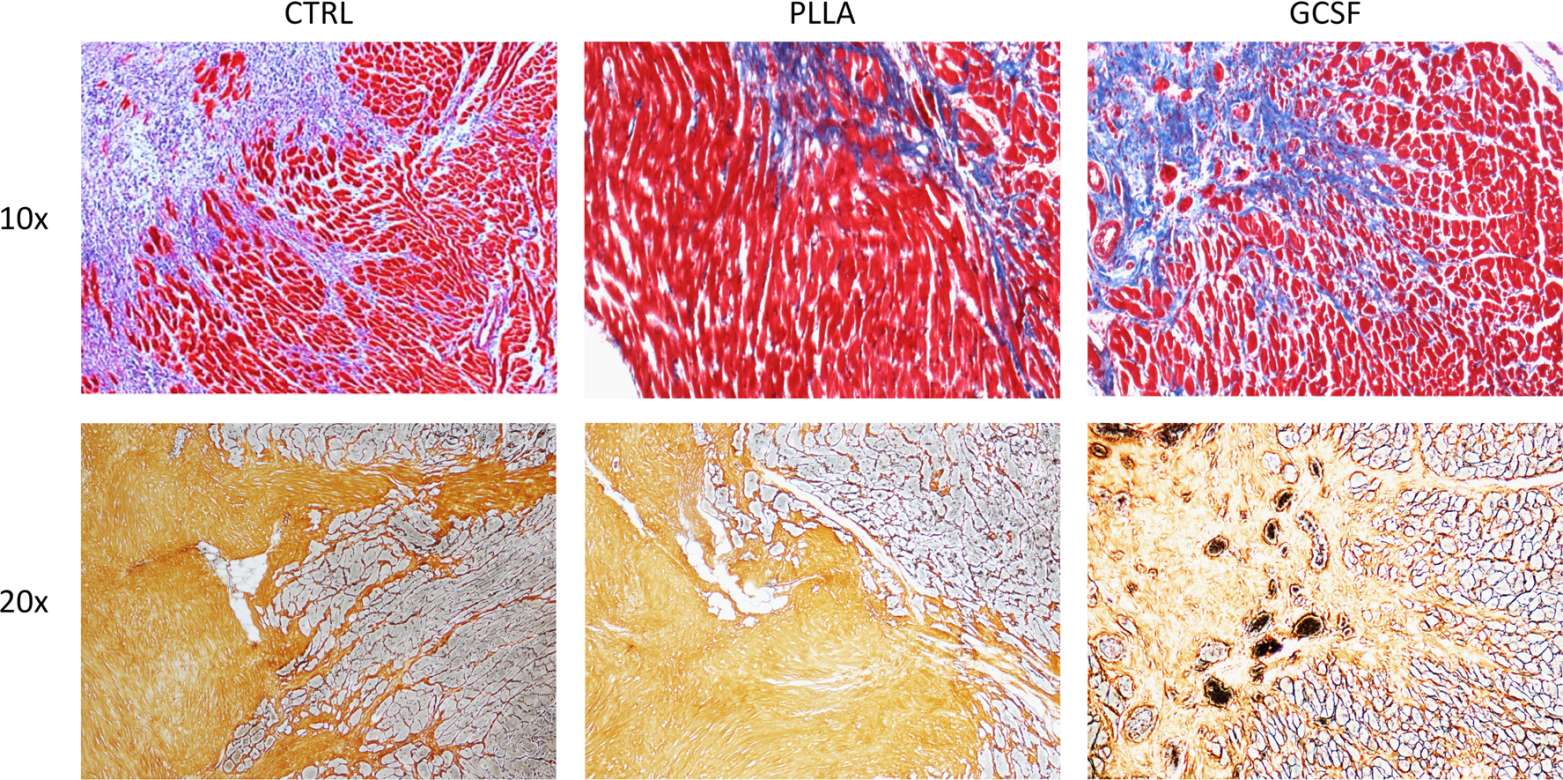
Qualitative assessment of ECM with Masson’s (*upper panels*) and Silver staining (*lower panels*) of the infarcted area. Denser, thicker, and scarcely organized collagen bundles are found in the MI/CTRL and PLLA/CTRL group at the level of the scar, while the PLLA/GCSF was characterized by a looser reticular organization of the collagen which appeared interspread among several neovessels

**Fig. 10:**
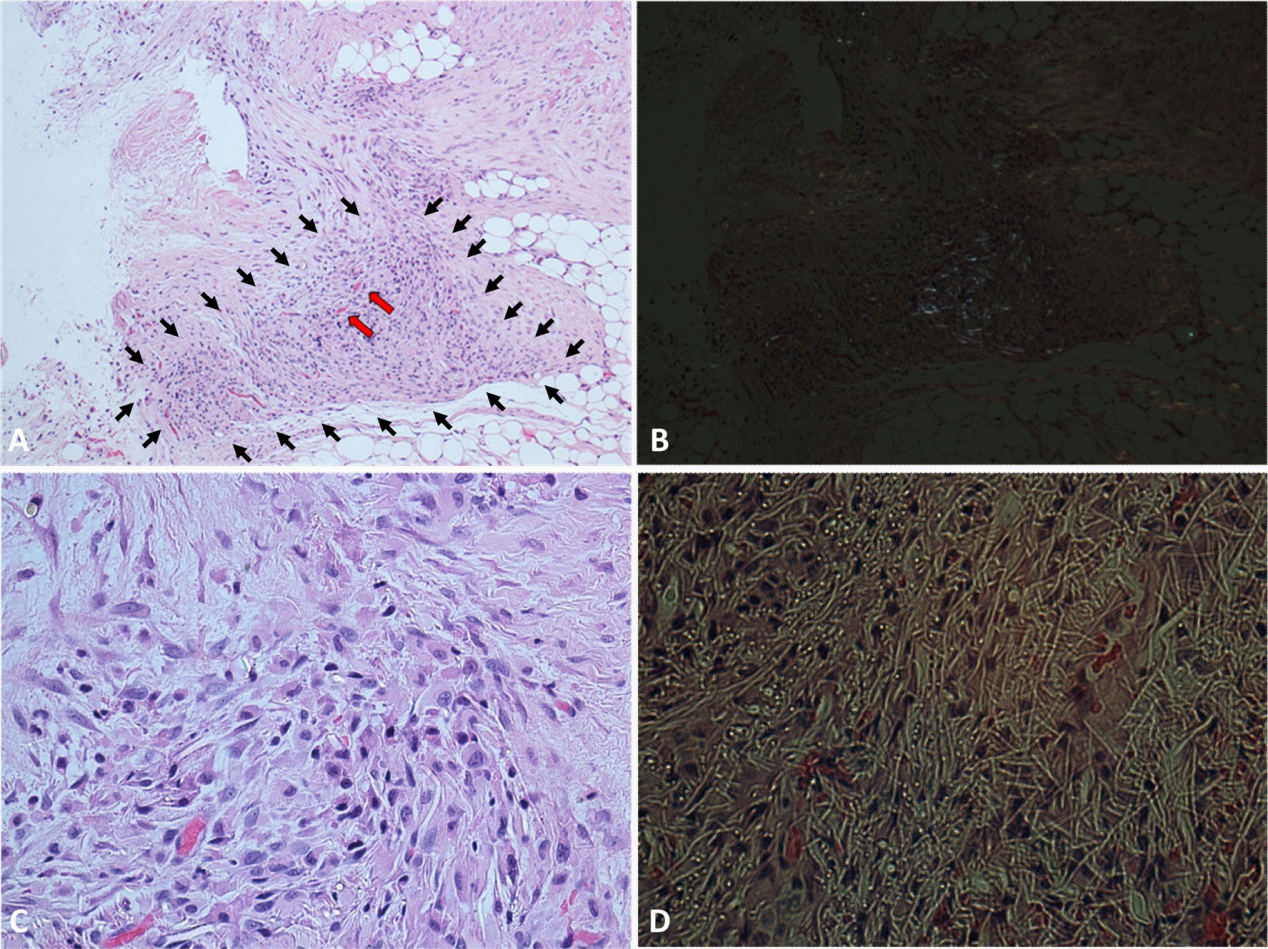
PLLA/GCSF patch in its site of implantation (grossly marked with *black arrows*, because of the partial integration with myocardial tissue), with evidence of angiogenesis (*red arrows*) (**a** H/E staining, magnification ×10). Phase-contrast image with specific polarized light, showing the bifrangent polymer (**b**). Magnifications of **a** (×40) (**c**) and **b** (×40) (**d**)

**Fig. 11:**
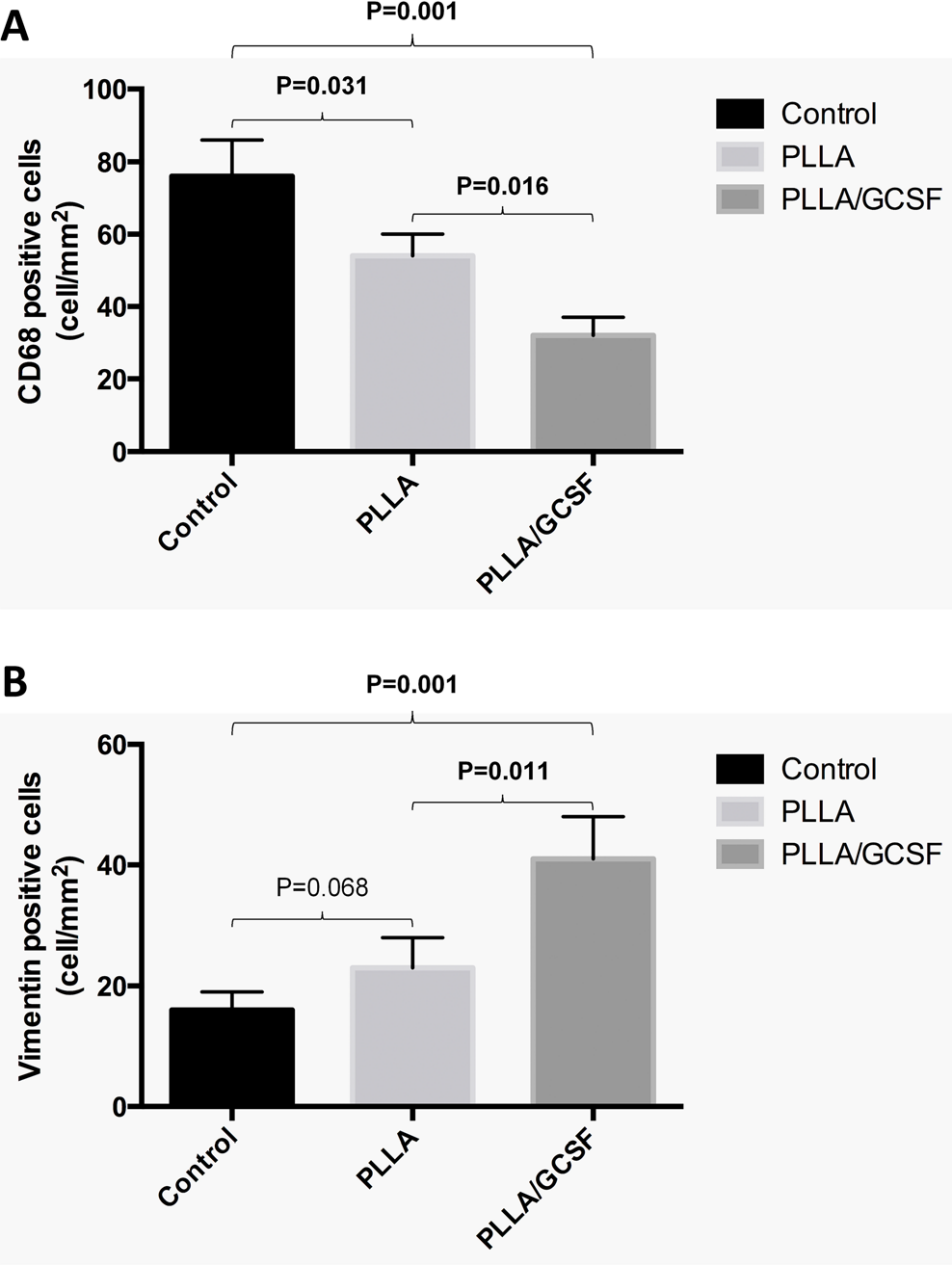
Count of CD68-positive (**a**) and vimentin-positive cells (**b**) among the three groups

**Fig. 12:**
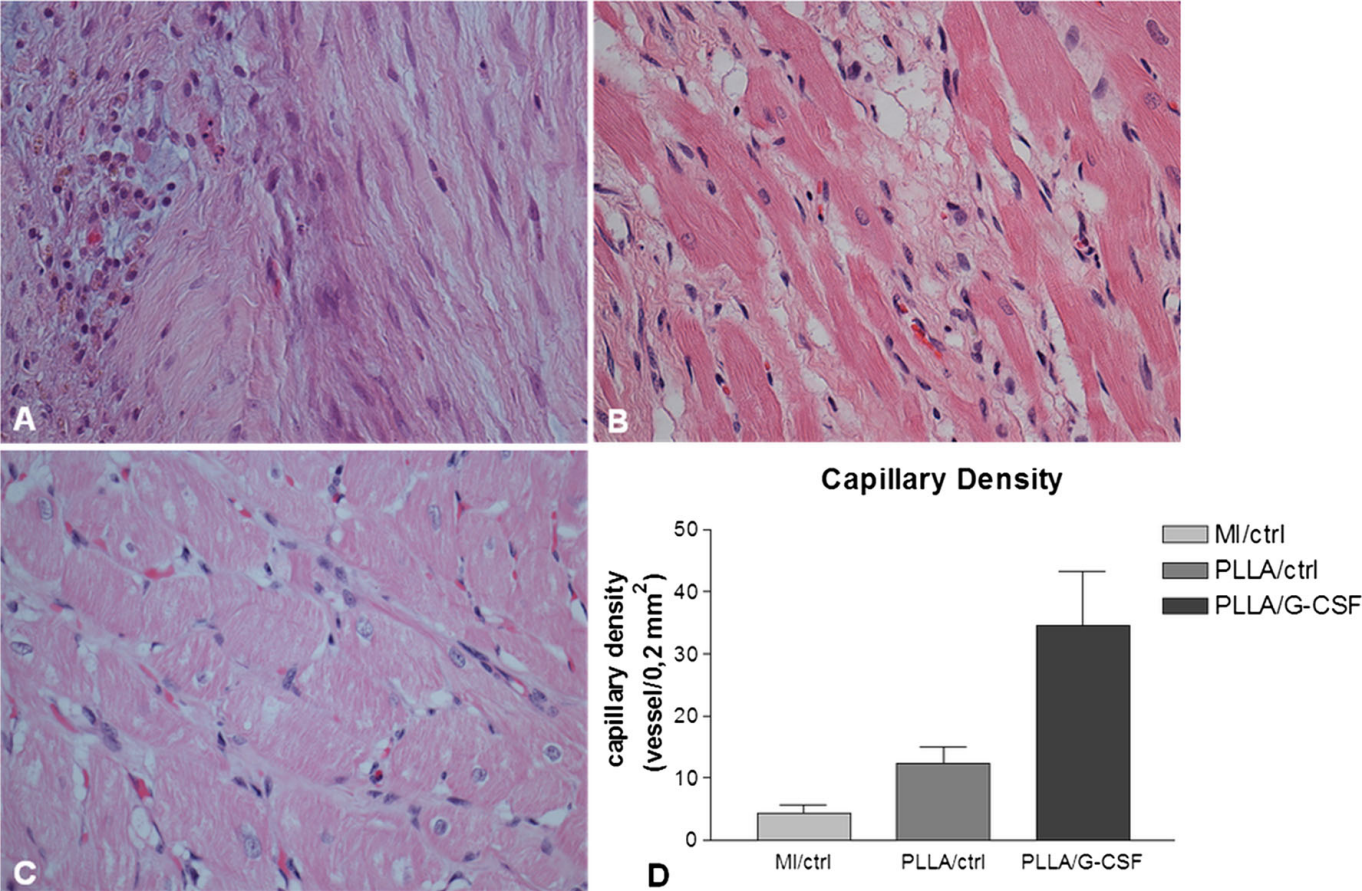
Capillary density measurement (magnification ×40) in MI/CTRL (**a**), PLLA/CTRL (**b**), and PLLA/GCSF (**c**) groups, with quantitative evaluation (**d**). Capillary density measurement showed a significant increase of neovessels in the PLLA/GCSF group compared to PLLA/CTRL (*p* value <0.01). Similarly, in the PLLA/CTRL group versus MI/CTRL group (*p* value <0.01)

**Fig. 13:**
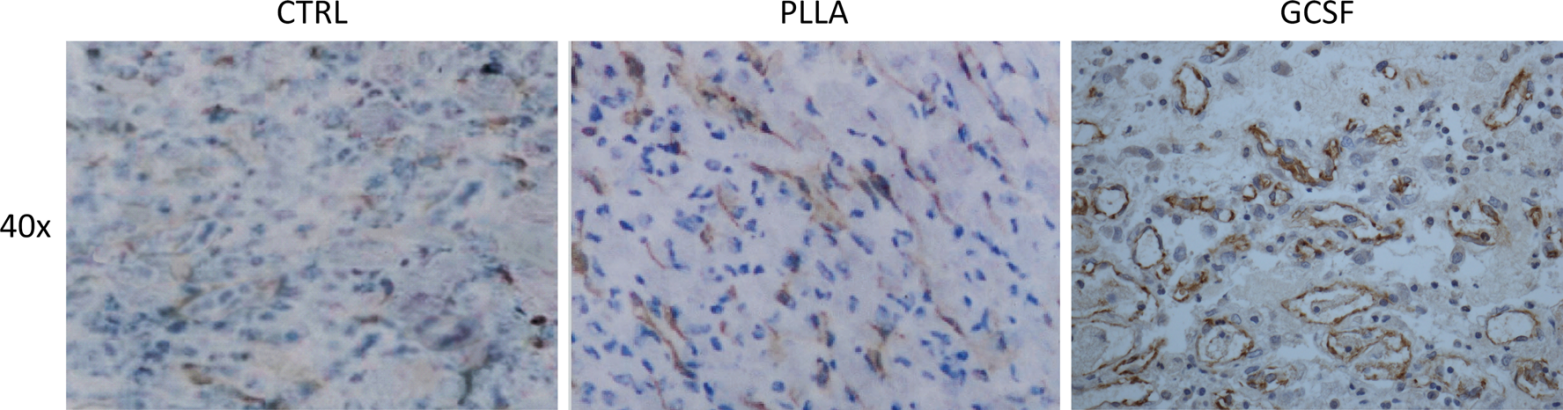
Immunohistochemistry for CD31-positive cells, indicating a higher degree of neoangiogenesis in PLLA/GCSF group

Hematoxylin-eosin staining in control chronic MI showed a cicatricial region characterized by cellular disarray and caotic deposition of extracellular matrix. Myocardial tissue showed typical features of chronic infarction with nuclear pyknosis, lipofuscin deposition, and intercellular calcifications. In the PLLA/GCSF group, the reduction of scar tissue is accompanied by an increase in cellularity and by a more organized fiber deposition, resulting in more loose collagen with fewer calcifications and increased angiogenesis (Figs. 8 and 9). Qualitative assessment of ECM with Masson’s and Silver staining showed denser, thicker, and scarcely organized collagen bundles in the MI/CTRL and PLLA/CTRL group at the level of the scar, while the PLLA/GCSF was characterized by a looser reticular organization of the collagen which appeared interspread among several neovessels (Fig. 9). With the aid of a light polarizator and in virtue of polymer refrangency, it was possible to study the cellular polymeric fiber relation within the patch (Fig. 10). Also, the PLLA/GCSF patch appeared cellularized with elements of different morphology and derivation. On the other hand, in the PLLA/CTRL group, the polymer appeared poorly cellularized although several inflammatory elements could be detected at the interface between the polymer and the native tissue. Differential count of CD68-positive cells in MI/CTRL, PLLA/CTRL, and PLLA/GCSF showed a statistically significant higher number of inflammatory cells in the control group and also in the nonfunctionalized scaffold in respect to the GCSF-releasing scaffold (Fig. 11). This finding was coupled with a statitically significant increase in the number of noninflammatory, fibroblast-like vimentin-positive cells in the GCSF group (Fig. 11). Capillary density measurement showed a significant increase of neovessels in the PLLA/GCSF group compared to PLLA/CTRL. Similarly, a signifcant increase in the number of neovessels could be observed in the PLLA/CTRL group in respect to MI/CTRL group (Fig. 12). Use of specific markers of angiogenesis (CD31) allowed to confirm the neoformation of the vascular structures observed (Fig. 13).

## Discussion

The development of functional bioengineered human cardiac tissue for the treatment of ischemic heart disease is a tremendous challenge for researchers and physicians. Many scaffold-based and scaffold-free approaches have been developed over the years, using natural biological materials such as collagen or fibrin, decellularized heart matrix, or synthetic polymers [34–37]. Some approaches rely on thin patches with well-aligned and electrically coupled cardiomyocytes, while cell-containing scaffolds are more easily vascularized by the host’s circulation. Patches can also be functionalized for the codelivery of peptides or other molecules promoting cell survival, control of inflammation, or improving cell function as a therapeutic modality for patients with ischemic heart disease [35].

The results of this preliminary study showed that (1) a PLLA-based electrospun scaffold efficiently integrated into a chronic infarcted myocardium without eliciting a strong inflammatory response; (2) functionalization of the biopolymer with GCSF led to a greater degree of cellular colonization within micrometric fibers in comparison to a nonfunctionalized scaffold; (3) PLLA/GCSF polymer induced a angiogenetic process with a statistically significant increase in the number of neovessels in respect to the nonfunctionalized scaffold; (4) PLLA/GCSF implanted at the level of the infarcted zone induced a reorganization of the ECM architecture leading to connective tissue deposition and scar remodeling; and (5) these findings were coupled with a statistically significant reduction in ESV and EDV, indicating an inhibitory effect of the scaffold on ventricular dilation, and an improvement in cardiac performance.

GCSF might play a pivotal role in ischemic cardiac remodeling with potential tremendous implication in future clinical practice. GCSF was found to be associated with antiapoptotic effect and inhibitory action on ventricle remodeling [8]. These effects are thought to be dependent on the receptor responsible for cardiac hypertrophy and via Akt pathway in failing ventricle after MI [38]. Recently, Rosenberg et al. demonstrated that the interaction between BM-derived stem cells and cardiomyocytes leads to a modified gene expression and induction of antiapoptotic pathways via a paracrine activation of Akt signaling [39]. These findings may at least in part explain the cardioprotective effects of stem cell therapy. Interestingly, Baldo et al. showed that GCSF reduces infarct size, preserves cardiac function, and prevents post-ischemic heart failure development in rats by an increase in Bcl-2 and Bcl-xL protein expression, eventually leading to an attenuation of apoptosis in the damaged tissue [40].

Also, GCSF activates the Wnt and Jak2 signals in cardiac cells leading to upregulation of connexin 43 (Cx43) protein expression and increasing its localization on the plasma membrane [41]. Cx43 is the major constituent of cardiac gap junction complex and is crucial for the regulation of the electrical properties of the heart reducing susceptibility to lethal arrhythmia after MI [42, 43]. In an in vivo small animal model of MI, GCSF treatment was shown to prevent ventricular arrhythmia associated to MI, reduce the duration of sustained ventricular tachycardia and improve survival [41]. To this extent, Baldo et al. have very recently suggested that GCSF might exert a direct stabilizing electrophysiological effect on infarcted myocardium independently of its genomic effects; in fact, its administration before coronary ligature in rats reduces the incidence of ventricular premature beats and ventricular tachycardia also in the following 30 min after MI occurrence [44]. Additionally, GCSF was found to enhance effects on angiogenesis [45], to induce in macrophages the synthesis of matrix metalloproteases (MMPs) that normally brings to adverse remodeling of ECM [46, 47], and to stimulate differentiation of muscular precursor cells to myotubes [48]. In light of these findings, several experimental efforts have been conducted to understand the molecular mechanism underlying GCSF effects and to optimize its in vivo and clinical use [49]. To prevent repeated drug administration and potentially associated complications, considering the 3.5-h half-life of GCSF, different solutions have been developed. Local injection of growth factor encapsulated hydrogels into skeletal muscle was gradually replaced by innovative metastable composite poly(ethylene glycol)-based hydrogel, obtaining a useful tool for controlled and sustained GCSF delivery and yielding extended mobilization of mononuclear cells and expanded CD34+ and CD31+ endothelial progenitor cells (EPCs) in vivo for up to 4 days [50]. However, even if controlled and sustained, the systemic delivery of cytokines or growth factors for regenerative purposes has shown inconsistent results in clinical trials and is also accompanied by numerous safety concerns and side effects [51].

Clearly, cardiac regeneration requires a complex cascade of events that cannot underestimate the structural and functional importance of the cardiac tissutal niche, along with factors and biological changes occurring in the microenvironment of the injured myocardium beyond the cellular counterpart. A real regenerative medicine approach should consider the significance of the ECM and the strong biological and physical signals that it can provide and receive [10]. The connective tissue atmosphere and the structural microenvironment in which cells are embedded affect their function and support their proliferation and differentiation. The creation of suitable structures (scaffolds), where the correct biochemical signals guide the growth of functional neotissue and provide an appropriate surface for cell attachment, proliferation, differentiation, and migration, appears crucial in this scenario [5, 52]. Tissue engineering (TE) approaches allowing for manufacturing of scaffolds tailored to promote and enhance tissue repair and regeneration are therefore emerging as a valid alternative. Scaffolds of poly-ethyl-acrylate and the self-assembling peptide gels are known to improve the uniformity and density of cell colonization in 3D structure [53], and this technology was previously found to be effective in engineering a functional heart muscle in vitro [54]. Another useful biofabrication technique is represented by electrospinning which enables to manufacture fibrillary matrix with ultrastructural properties featuring in micrometric scale and thus closely simulating the native ECM histoarchitecture [17]. In our group, we focused on the development of ECM-mimicking scaffolds for cardiac tissue engineering purposes and a GCSF-functionalized scaffold has been produced by electrospinning [16].

The biological and mechanical properties of the scaffold used in this study have been previously described in other work from our group [16]. To overcome many of the biological, economical, logistic, and ethical concerns which are currently known to hurdle the clinical application of cellular therapy, we decided to explore a strategy that avoids the use of cells but mostly relies on the biomimetic design of the scaffold and its paracrine effect as a “drug-eluting device,” and might therefore be introduced in clinical practice more rapidly. With this in mind, and differently from other published approaches [55, 56] based on the application of tissue-engineered constructs in an acute context immediately after the experimental generation of a MI, we used a chronicized stabilized MI model infarction which reflects the normal conditions routinely encountered by surgeons in the clinical scenario. In light of this translational vocation, the scaffold was not pre-seeded with stem cells before implantation as was the case for other described approaches [55, 56]. Tissutal resident stem cells and progenitor cells known to inhabit specific niches in the heart [57, 58], or bone marrow-derived stem cells recruited after a myocardial injury could therefore colonize an artificial matrix that, for its internal structure, closely simulates the native ECM atmosphere and actively emanates biological signals to support their survival and differentiation. This 3D ECM-like environment would therefore promote and boost the endogenous reparative process triggered by organ damage.

In our setting, the scaffold, by mimicking the ECM histoarchitecture, provided at the same time a biological and mechanical support during the healing process, exerting a modulatory effect on cell colonization, and guiding and accompanying the body regenerative response. Noteworthy, our findings are consistent with a more controlled and modulated tissutal reaction in the PLLA/GCSF group, as testified by the reduced presence of inflammatory cells in this group. The biological signaling arising from the scaffold could mitigate the host response reducing the intensity of the inflammatory phase of the healing process (as confirmed by both radiological and histological observations) and orientate cellular reaction toward effective regeneration. The biomimetic principle inspiring the structure of this scaffold, along with the functionalization with bioactive molecules, allowed to guide the reparative processes and modulate the microenvironment of the damaged tissue favoring the regenerative drive over the inflammatory reaction.

GCSF-induced angiogenesis is well known in the literature [59–62], but in this setting, it occurred in the context of the implanted scaffold. Histological analysis showed a significant number of neovessels within the scaffold framework. The biopolymer provided both stimuli and structural support to angiogenesis, guiding this process also from the spatial and topographical point of view. This finding could achieve an important significance when considering the clinical incidence of situations in which small coronaries caliber or scarcity of collateral flow in myocardial zones involved by ischemic injury impede to perform coronary artery bypass (CABG) [63]. In situ-guided angiogenesis through the use of tissue-engineered drug-releasing scaffolds might be an attractive alternative to create a “neoangiogenetic bypass” in myocardial regions which cannot be revascularized, as an adjunct to routinely used autologous grafts.

In our study, neoangiogenesis was associated to significant increase in cardiac performance as testified by measurements of EF, stroke volume, and cardiac output along with the fraction of shortening of the LV. Also, we found a significant reduction of EDV and ESV, reliably indicating that the benefit on cardiac performance can be due to a restraint action exerted by the biopolymer engrafted in the myocardial wall. This finding might be reliably explained by a phenomenon of compensatory hypertrophy induced by the biomaterial, or, alternatively, by a significant cellular colonization of the patch leading to production of new extracellular matrix. However, the reshaping effect induced by the surgical implant of the patch itself with consequent reduction of LV cavity could invalidate this hypothesis. Nevertheless, the improvement in LV volumes has been found in the PLLA/GCSF group only, while both the MI/CTRL and the PLLA/CTRL group showed a progressive dilation of LV chambers. Therefore, the reshaping effect observed in PLLA/GCSF group is unlikely to be due to a “suction cup” effect. Clearly, further investigations are required to elucidate the mechanisms underlying these findings.

In the PLLA/GCSF group, a dense cellularization with subsequent organization in a neotissue was observed histologically, while the PLLA group showed a scarce cellular colonization. This finding is in line with the known chemotactic effect of GCSF; however, the immunological analysis of the origin of the cells populating the scaffold can be useful to explain these histological observations. However, the inflammatory reaction was not exuberant indicating a good degree of biocompatibility of the material within the host tissue. Differential cell count for inflammatory and noninflammatory cells showed a stastistically significant lower number of inflammatory element in the GCSF group. In parallel, vimentin-positive cell number was higher in the functionalized scaffold group suggesting a shift in the ratio between inflammatory and fibroblast-like cells. Taken together, these finindings support the mentioned idea of a more modulated inflammatory response accompanied by an active reparative response. Athough we mainly used vimentin as a fibroblastic marker to investigate ECM neoformation, Odorfer et al. and parallely Antonistitis et al. intriguigly used this protein as a marker of early differentiated cardiomyocites [31, 64]. Despite in need of confirmatory study, the increased presence of early differentiated cardiomoycites in the PLLA/GCSF scaffold might open novel fascinating avenues in in situ tissue engineering and fuel further research on this field.

Along with this finding, the imaging analysis through CT demonstrated a significant increase in the ventricular mass in the PLLA/GCSF group. Beside the caveat of the low sample size and the resolution and technical limits of the imaging system used, this data might confirm the histological observation of the constitution of neotissue or might suggest a phenomenon of a compensatory LV hypertrophy induced by the biopolymer. In the latter hypothesis, we could speculate that GCSF with its known antiapoptotic effects might have positively influenced the delicate balance between apoptosis and hypertrophy known to characterize post-MI ventricular remodeling.

Our results are similar to the ones by Zhang’s group, who used a fibrin patch to enhance production and delivery of cytokines from vascular cells for myocardial repair. In vitro experiments proved that the functionalized patch reduced myocyte apoptosis and contractile dysfunction, and that patch-enhanced cell therapy was associated with a reduced infarct size and enhanced angiogenesis 4 weeks after MI [65, 66]. However, these results were obtained using a cell-based approach in which progenitor cells delivered by a fibrin patch and liberating a plethora of biochemical signals induced angiogenesis and improvement in cardiac function. Despite using different “myocardial enhancers,” those similar results suggest that many cofactors might ultimately convey into a unique—unknown—final mechanism of action which triggers tissue repair, and that the use of smart ECM-mimicking biopolymers delivering the correct sequence of biological signaling might produce similar benefit without the addition of exogenous cells sources. Future studies are warranted to elucidate this point and provide a comprehensive view of these phenomena, but there are several studies in support of this hypothesis reporting approaches based on injectable polymerizing hydrogels exhibiting natural cellular adhesion sites and capable of reproducing the mechanical properties of the nanofibrous network of the ECM [67]. In in vivo studies, these injectable polymers were able to increase LV myocardial wall thickness, limiting the LV remodeling process and preserving myocardial contractility [68].

Despite preliminary and purely speculative in its nature, this study is demonstrating the effectiveness of a GCSF-releasing polymer in a model of chronic myocardial infarction. Differently from the current paradigm in TE and cardiovascular regenerative medicine, the described approach does not imply the use of exogenous stem cells but relies on the optimization of the biomaterial design and on its biofunctionalization with the aim to create a device able to simulate the native histoarchitecture of the cardiac ECM and modulate the microenvironment of the infarcted myocardium through the release of growth factors. The biomaterial engrafted in the host tissue would emanate a biological signal able to stimulate the reparative process and orientate the regenerative potential and the differentiation of endogenous stem cell resources resident in the heart or migrated from other organs.

Almost 10 years ago, the pioneering works of Chachques and Carpentier on collagen-based sponges for myocardial repair culminated in the Magnum trial which evaluated the effect of cell-seeded collagen scaffolds on the myocardial scar in patients undergoing coronary surgery, but this approach still included the use of autologous mononuclear bone marrow cells (BMCs) with the economical and logistic issues associated to cell therapy [69]. However, recent meta-analysis of randomized clinical trials concluded that intracoronary autologous BMC treatment, which is more practical and technical feasible than the patient-specific fabrication of a living myocardial construct, led to an improvement in EF and ameliorated ventricular remodeling over time after implantation. Those results are associated with reduced hospital admission for heart failure, recurrent myocardial infarction, or angina [70, 71]. In front of these evidences and if a “stem cell-free approach” is not successful, a challenging perspective could be therefore the evaluation of intravascular autologous peripheral stem cell administration, used as a “burst therapy”, combined with the local application after myocardial revascularization of functionalized scaffolds able to provide a sustained delivery of growth factors and a platform to recruit and locally capture the stem cells injected. By conveying various effects, myocardial perfusion could be enhanced by the positive remodeling due to the cellular and ECM-surrogate interplay.

### Limitations

Among the limitations of this study, authors acknowledge the lack of a cytofluorimetric and immunophenotypic analysis of the cells colonizing the scaffolds and in blood stream to demonstrate the chemotactic effect of GCSF. The scarcity of specific antibody to detect rabbit markers limited this study. Secondly, a more detailed quantitative and qualitative analysis of the neomatrix (collagen type I, collagen type III elastin, etc.) deposited within the biopolymer to support the findings on the geometrical remodeling of the LV is lacking. Similarly, apoptosis and proliferation analysis along with an evaluation of the presence of neoangiogenetic factors such as VEGF or bFGF might be useful to address questions on induced LV hypertrophy. Considering the known effect of GCSF in the upregulation of Cx43 and prevention of malignant ventricular arrhythmia, an immunological analysis of the expression of this marker might be advocated although no arrhythmias were registered at ECG throughout the study. These parameters are currently under evaluation in our group.

Another limitation relies in the lack of quantitation of the potential GCSF release from the patch in to the systemic circulation. In this study, in order to avoid biases related to the presence of connective tissue in the epicardium, which might have affected the results and also the diffusion of the molecules, we scarified the infarcted region before implantation to guarantee a direct contact of the patch with the myocardial muscle and microvessels. GCSF diffusion was analyzed and characterized in our previous foundation study. However, the scarcity of specific antibody to detect rabbit markers prevented to perform an actual quantitative assay (i.e., ELISA) to measure systemic GCSF released by the scaffold. However, based on our previous analysis in vitro [16], we might reliably speculate that the actual amount reaching the circulation is negligible and that the functional activity was exerted locally.

Finally, despite the sample size has been calculated using reverse power analysis on the bases of previously published reports in this field, a larger number of observations might be required to confirm the findings here described.

### Clinical Significance

A GCSF-functionalized PLLA scaffold implanted on an infarcted zone induced a neoangiogenetic response and a reorganization of the ECM architecture leading to connective tissue remodeling with a positive impact on ventricle remodeling and performance. Scaffold was not pre-seeded with stem cells but was used as a “smart” biomaterial with the rationale to boost the endogenous reparative response exploiting the biomimetic design of the scaffold and the biological signaling emanated. Indeed, the biomaterial was able to stimulate and modulate the physiological process of repair in the absence of exogenous sources of cells. This might indicate its potential for a use in the clinical scenario as an “off-the-shelf” device, circumventing the logistical economical and ethical issues related to stem cell therapy. In light of this translational inspiration, the animal model was selected to simulate as closely as possible the clinical conditions encountered by surgeons in the routine practice. Additionally, the finding of local enhancement of angiogenesis in the surrounding area of an infarct could also achieve an important significance when considering the incidence of situations in which small coronaries caliber or scarcity of collateral flow in ischemic myocardial zones impede to perform CABG. In situ-guided angiogenesis by tissue-engineered drug-releasing scaffolds might be an attractive alternative to create a “neoangiogenetic bypass” in myocardial regions which cannot be revascularized, as an adjunct to routinely used autologous grafts. This study represents the first pre-clinical experience proving a role for PLLA/GCSF scaffold in myocardial infarction and, if confirmed by further investigations, might support the introduction of such scaffolds into the clinical scenario in patients undergoing surgical myocardial revascularization.

## Abbreviations

BM: Bone marrow
CO: Cardiac output
ECM: Extracellular matrix
EDD: End-diastolic diameter
EDV: End-diastolic volume
EF: Ejection fraction
EPC: Endothelial progenitor cells
ESD: End-systolic diameter
ESV: End-systolic volume
FE-SEM: Field emission scanning electron microscopy
FS: Fraction of shortening
GCSF: Granulocyte colony-stimulating factor
LV: Left ventricle
LVM: Left ventricle mass
MI: Myocardial infarction
PLLA: Poly-l-lactide
PS: Peak stress
SF: Strain at failure
SV: Stroke volume
TE: Tissue engineering
TECG: Tissue-engineered cardiac graft
